# How to obtain the image-derived blood concentration from ^89^Zr-immuno-PET scans

**DOI:** 10.1186/s40658-024-00621-7

**Published:** 2024-02-07

**Authors:** Jessica E. Wijngaarden, Amina Ahbari, Johanna E. E. Pouw, Henri N. J. M. Greuter, Idris Bahce, Gerben J. C. Zwezerijnen, Daniëlle J. Vugts, Guus A. M. S. van Dongen, Ronald Boellaard, C. Willemien Menke-van der Houven van Oordt, Marc C. Huisman

**Affiliations:** 1 Department of Radiology and Nuclear Medicine, Amsterdam UMC Location Vrije Universiteit Amsterdam, De Boelelaan 1117, 1081 HV Amsterdam, The Netherlands; 2 Cancer Center Amsterdam, Imaging and Biomarkers, Amsterdam, The Netherlands; 3 Department of Medical Oncology, Amsterdam UMC Location Vrije Universiteit Amsterdam, Boelelaan 1117, Amsterdam, The Netherlands; 4 Department of Pulmonary Medicine, Amsterdam UMC Location Vrije Universiteit Amsterdam, Boelelaan 1117, Amsterdam, The Netherlands

**Keywords:** ^89^Zr-immuno-PET, Blood sampling, Image-derived blood concentration, Monoclonal antibodies

## Abstract

**Background:**

PET scans using zirconium-89 labelled monoclonal antibodies (^89^Zr-mAbs), known as ^89^Zr-immuno-PET, are made to measure uptake in tumour and organ tissue. Uptake is related to the supply of ^89^Zr-mAbs in the blood. Measuring activity concentrations in blood, however, requires invasive blood sampling. This study aims to identify the best delineation strategy to obtain the image-derived blood concentration (IDBC) from ^89^Zr-immuno-PET scans.

**Methods:**

PET imaging and blood sampling of two ^89^Zr-mAbs were included, ^89^Zr-cetuximab and ^89^Zr-durvalumab. For seven patients receiving ^89^Zr-cetuximab, PET scans on 1–2 h, 2 and 6 days post-injection (p.i.) were analysed. Five patients received three injections of ^89^Zr-durvalumab. The scanning protocol for the first two injections consisted of PET scanning on 2, 5 and 7 days p.i. and for the third injection only on 7 days p.i. Blood samples were drawn with every PET scan and the sample-derived blood concentration (SDBC) was used as gold standard for the IDBC. According to an in-house developed standard operating procedure, the aortic arch, ascending aorta, descending aorta and left ventricle were delineated. Bland–Altman analyses were performed to assess the bias (mean difference) and variability (1.96 times the standard deviation of the differences) between IDBC and SDBC.

**Results:**

Overall, the activity concentration obtained from the IDBC was lower than from the SDBC. When comparing IDBC with SDBC, variability was smallest for the ascending aorta (20.3% and 17.0% for ^89^Zr-cetuximab and ^89^Zr-durvalumab, respectively). Variability for the other regions ranged between 17.9 and 30.8%. Bias for the ascending aorta was − 10.9% and − 11.4% for ^89^Zr-cetuximab and ^89^Zr-durvalumab, respectively.

**Conclusions:**

Image-derived blood concentrations should be obtained from delineating the ascending aorta in ^89^Zr-immuno-PET scans, as this results in the lowest variability with respect to sample-derived blood concentrations.

**Supplementary Information:**

The online version contains supplementary material available at 10.1186/s40658-024-00621-7.

## Introduction

Positron emission tomography (PET) imaging allows visualization and quantification of radiolabelled molecules (tracers) in vivo. PET imaging of zirconium-89 labelled monoclonal antibodies (^89^Zr-mAbs), known as ^89^Zr-immuno-PET, enables the assessment of mass dose selection, pharmacokinetic properties of the drug, and efficacy and toxicity of immunotherapy [1].

The uptake of tracers in tumours and healthy tissue is commonly quantified using the standardized uptake value (SUV). The SUV assumes that the plasma clearance of the tracer is comparable between patients, conditions and administered mass doses [2]. However, studies have shown that ^89^Zr-mAb pharmacokinetics differ between mass doses, affecting the reliability of the SUV [3, 4]. Correction of the tumour activity concentration for the blood activity concentration, i.e. tumour-to-blood ratio (TBR), has been postulated to provide more reliable measurements of ^89^Zr-mAb uptake (see [5] for a similar approach for [^18^F]-FDG).

The activity concentration in blood can accurately be measured by taking blood samples from the patient at the imaging time points. However, blood sampling is an additional and invasive procedure which may cause discomfort for the patient. Furthermore, missing blood samples would lead to missing data points for TBR. Even more so, blood sampling is often not included in the study protocol. As an alternative to blood sampling, the activity concentration in blood can also be derived from the PET scan [6]. This is commonly known as the image-derived input function (IDIF) when obtained during dynamic PET imaging. However, in this study it is referred to as the image-derived blood concentration (IDBC), since single observations were evaluated instead of the activity concentration over time.

The use of IDIF has been studied previously for different tracers and blood pool regions. In [^18^F]-FDG PET imaging, the IDIF obtained from the ascending aorta showed the best agreement with online arterial sampling [7, 8]. IDIFs have also shown to be suitable as replacement for online arterial sampling of several tracers used in brain PET imaging [9]. There are differences between ^18^F and ^89^Zr which may influence quantification. The positron yield of ^89^Zr is 23%, compared to 97% for ^18^F [10], and for that reason and the long physical half-life of ^89^Zr, less ^89^Zr-labelled antibody is typically injected to keep radiation exposure within safety limits. The result is a scan with a lower signal-to-noise ratio and therefore a higher variability for ^89^Zr-PET quantification. Additionally, ^89^Zr has a longer positron range which decreases the spatial resolution [10]. This increases partial volume effects (PVE), an imaging effect that causes underestimation of the activity concentration in a volume of interest (VOI) due to spill-out into surrounding tissue [11]. Therefore, before using IDBC for ^89^Zr-labelled tracers, analyses of different blood pool regions compared to blood sampling needs to be performed.

For ^89^Zr-immuno-PET, IDBC obtained from the left ventricle has previously been compared with sampled blood activity, showing a good agreement for patients with a body weight below 100 kg [12]. Additionally, Jauw et al. studied the noise-induced variability in IDBC obtained from the aortic arch and proposed to investigate different blood pool regions to optimize the delineation method [13]. To our knowledge, the use of IDBC has not yet been compared between multiple blood pool regions for ^89^Zr-immuno-PET studies.

This study evaluates the best delineation strategy to obtain the image-derived blood concentration (IDBC) from a ^89^Zr-immuno-PET scan.

## Methods

### Study protocols

PET imaging and venous blood sampling data of two clinical ^89^Zr-immuno-PET imaging studies were analysed. For seven patients receiving 500 mg/m^2^ unlabelled cetuximab with 10 mg 37 MBq ^89^Zr-cetuximab (NCT02117466), whole-body PET/CT scans (Philips Gemini or Ingenuity) were acquired at 1–2 h, 2 and 6 days p.i. Five patients received three injections of 2 or 22.5 mg 37 MBq ^89^Zr-durvalumab (NCT03519971). The scanning protocol for the first two injections consisted of PET/CT scanning (Philips Gemini, Ingenuity or Vereos) on 2, 5 and 7 days p.i. and for the third injection on 7 days p.i. In total, 49 scans were included in the analysis. Blood samples were drawn at every imaging time point. Both studies were reviewed and approved by the Central Committee on Research Involving Human Subjects of the Netherlands and the Medical Ethics Review Committee of the Amsterdam University Medical Centers. All patients gave written informed consent prior to study participation.

### Delineation of blood pool regions

A standard operating procedure was developed and checked with a nuclear medicine physician for delineating the aortic arch, the ascending aorta, the descending aorta and the left ventricle. All four blood pool regions were delineated from an axial field of view based on the low dose CT (ldCT). The PET was used to correct for any mismatches between the ldCT and PET. The ascending and descending aorta were delineated on at least five consecutive slices and up to ten slices, until the end of the structure. Four regions of interest were placed in the aortic arch on two consecutive slices with a total volume of 4.6 mL. The delineations were ensured to be placed within the lumen. A sphere of 1.7 mL was placed in the centre of the left ventricle. The detailed procedure with examples can be found in Additional file 1: SM1.

### Statistical analyses

Pearson correlations were performed to assess the relation between the IDBC and the SDBC. Since correlations may be misleading in evaluating the agreement between two methods and the feasibility of replacing one method with the other, Bland–Altman analyses were performed as well [14]. The Bland–Altman plots show the percentage bias between the IDBC and the SDBC on the *y*-axis and the mean of the two methods on the *x*-axis. The percentage bias for each data point was calculated as: $\%{\text{bias}}= \frac{({\text{IDBC}}-{\text{SDBC}})}{({\text{SDBC}}+{\text{IDBC}})/2} \times 100\%$, where SDBC = blood sample and IDBC = image-derived blood concentration. The mean percentage bias was obtained to evaluate the accuracy of the IDBC compared to the SDBC. Subsequently, 1.96 times the standard deviation, was obtained to evaluate the variability between the IDBC and the SDBC.

Additionally, the biological half-life of the ^89^Zr-mAbs was estimated using both SDBC and IDBC, for the patients of whom blood samples and PET scans were obtained from at least three time points. Values for the half-life were obtained by fitting a linear model to the data in the form: ${\text{ln}}\left(BC\right)= -(\frac{{\text{ln}}\left(2\right)}{{t}_{1/2}})\times {\text{time}}$, where BC is the IDBC or SDBC [Bq/mL] and *t*_1/2_ is the half-life [h]. For calculation of the sample-derived half-life, only the samples obtained at the PET time points were included. Bland–Altman analyses were performed and three outliers were excluded based on values more than 1.96 times the standard deviation.

## Results

Overall, the activity concentration obtained from the IDBC was lower than from the SDBC. Strong statistically significant correlations (*r* > 0.99, *p* < 0.001) were found between IDBC and SDBC for both ^89^Zr-mAbs and all four blood pool regions (see Table 1). The corresponding regression plots for ^89^Zr-cetuximab and ^89^Zr-durvalumab are shown in Additional file 1: Figs. S1 and S2.

**Table 1:** Pearson correlations between IDBC and SDBC

Blood pool region	Intercept	Slope	*r*	*p* Value
^*89*^*Zr-cetuximab*				
Aortic arch	361	0.66	0.99	< 0.001
Ascending aorta	273	0.80	0.99	< 0.001
Descending aorta	300	0.67	0.99	< 0.001
Left ventricle	292	0.71	0.99	< 0.001
^*89*^*Zr-durvalumab*				
Aortic arch	288	0.74	0.99	< 0.001
Ascending aorta	38	0.88	0.99	< 0.001
Descending aorta	194	0.79	0.99	< 0.001
Left ventricle	− 53	0.94	0.99	< 0.001

Pearson correlation coefficients and significance values for correlations between IDBC and SDBC showed strong statistically significant correlations for both ^89^Zr-mAbs and all four blood pool regions

**Fig. 1:**
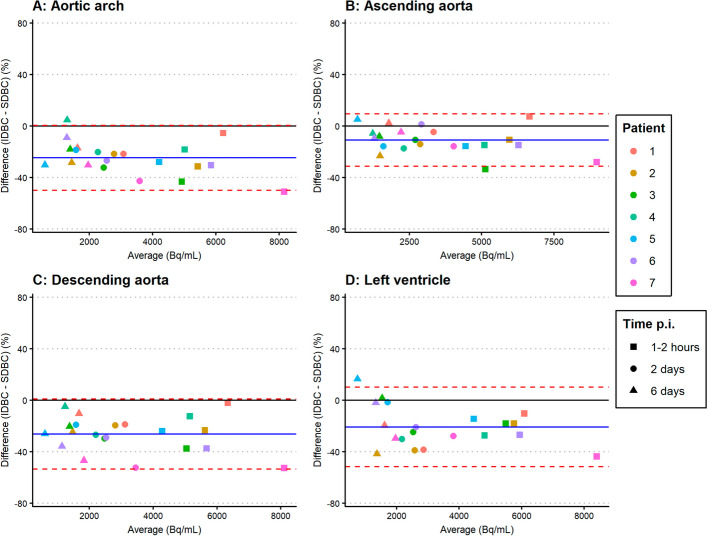
Bland–Altman plots showing the agreement between the IDBC and SDBC of ^89^Zr-cetuximab for the aortic arch (**A**), ascending aorta (**B**), descending aorta (**C**) and left ventricle (**D**). Data of seven patients and all time points are shown. The blue solid lines indicate the mean bias (mean difference), and the red dashed lines indicate the variability (1.96 times the standard deviation of the differences)

**Fig. 2:**
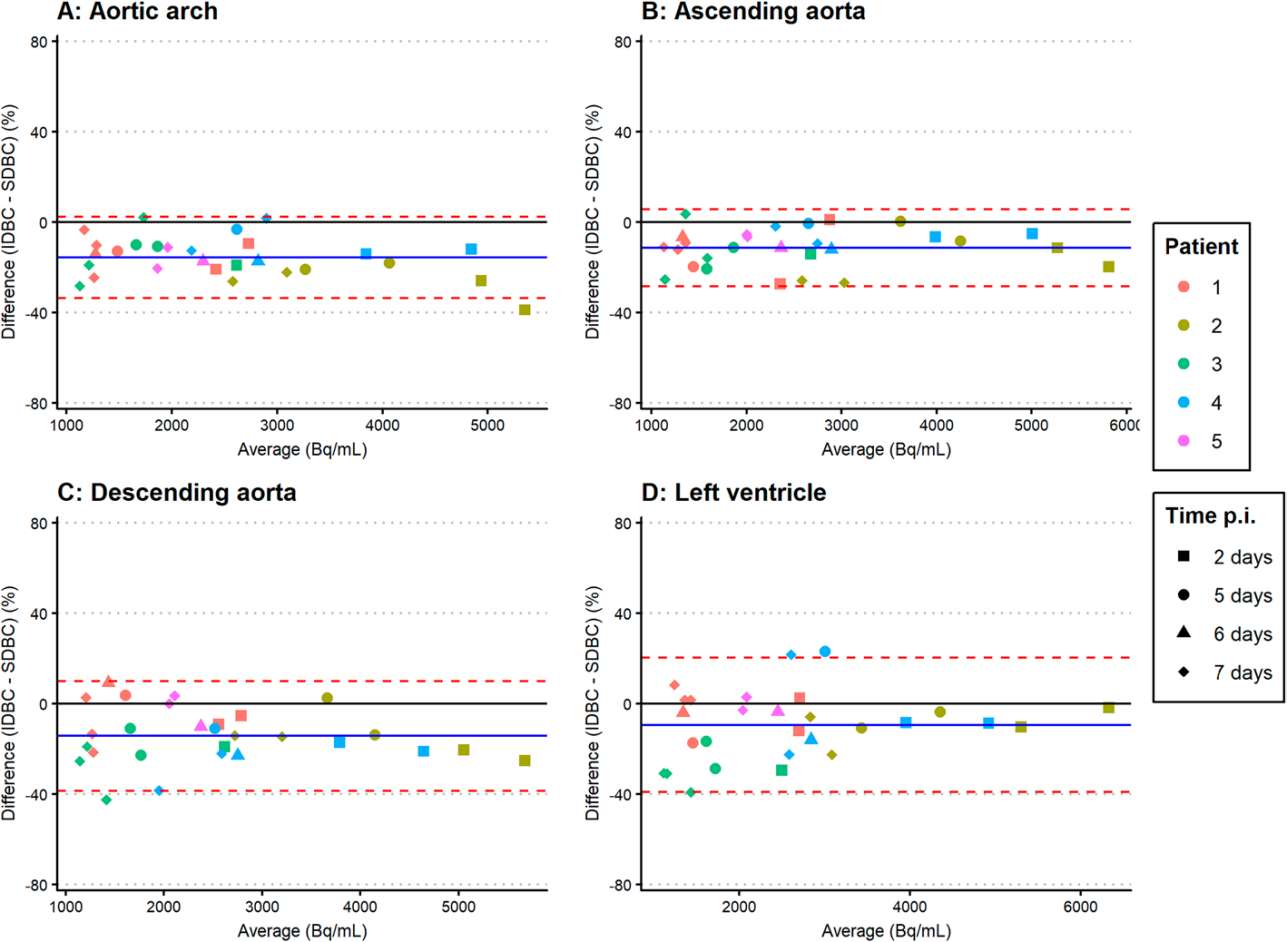
Bland–Altman plots showing the agreement between the IDBC and SDBC of ^89^Zr-durvalumab for the aortic arch (**A**), ascending aorta (**B**), descending aorta (**C**) and left ventricle (**D**). Data of five patients and all time points are shown. The blue solid lines indicate the mean bias (mean difference) and the red dashed lines indicate the variability (1.96 times the standard deviation of the differences)

Bland–Altman analyses comparing the IDBC with SDBC showed biases ranging from − 9.4 to − 26.3% and variability ranging from 17.0 to 30.8% (see Table 2, Fig. 1). For ^89^Zr-cetuximab, the ascending aorta showed the smallest bias (− 10.9%) and variability (20.3%). For ^89^Zr-durvalumab, variability was also smallest for the ascending aorta (17.0%). However, the smallest bias was found for the left ventricle (− 9.4%) with the ascending aorta as second best (11.4%) (see Table 2, Fig. 2).

**Table 2:** Bland–Altman analysis comparing IDBC with SDBC

Blood pool region	Mean percentage bias (%)	1.96 Times standard deviation (%)
^*89*^*Zr-cetuximab*		
Aortic arch	− 24.7	25.1
Ascending aorta	− 10.9	20.3
Descending aorta	− 26.3	27.2
Left ventricle	− 20.8	30.8
^*89*^*Zr-durvalumab*		
Aortic arch	− 15.7	17.9
Ascending aorta	− 11.4	17.0
Descending aorta	− 14.2	24.2
Left ventricle	− 9.4	29.6

Bias and variability in IDBC compared with SDBC for both ^89^Zr-mAbs and all four blood pool regions. The ascending aorta showed the best overall results in bias and variability

For all blood pool regions, the image-derived half-life could be obtained with a maximum bias of 10.9% compared to the sample-derived half-life. Overall, the ascending and descending aorta showed the lowest bias. An overview of the comparisons in half-life is shown in Additional file 1: Table S1.

## Discussion

The IDBC from all four blood pool regions was found to be strongly correlated with SDBC for ^89^Zr-immuno-PET studies. Overall, the ascending aorta showed the best agreement between the IDBC and SDBC as evaluated with Bland–Altman analyses.

The ascending aorta showed the lowest and similar variability for both ^89^Zr-mAbs. The differences in variability between the ascending aorta and the other regions might be due to the feasibility of the delineation. Delineation of the ascending and descending aorta was found to be most feasible practically, as experienced by the person delineating the regions. It was slightly more difficult to delineate within the lumen of the aortic arch, because of the curvature of the arch. The left ventricle was difficult to localize based on the ldCT, because the X-ray attenuation in the ventricle was similar to the surrounding heart tissue. Additionally, the left ventricle is more susceptible to movement of the heart. This may lead to misalignment of the PET and CT scans, and thereby inaccurate attenuation correction around the left ventricle. This would explain the smaller variability found for the ascending aorta compared to the aortic arch and left ventricle. The variability of 29.6% and 30.8% for the left ventricle found in this study was similar to the SD of 16.9% (which is about half of the RC) found in previous literature studying image-derived ^89^Zr-mAb concentrations in the left ventricle [12]. Previous literature on image-derived ^89^Zr-mAb concentrations in the aortic arch found similar results as well [13], with RC in the range of 17–43% compared to 18% and 25% found in this study. The Bland–Altman plots also show larger variability at lower activity concentrations, which are obtained at later time points. At later time points, PET scans contain much more noise because less radioactivity is left. This increases the uncertainty in the data, leading to higher variability.

The IDBC was lower than the SDBC indicated by the negative bias. The presence of the bias in our data suggests the usefulness of a procedure for cross-calibration of the well counter to the dose calibrator. In Additional file 1: SM2, we provide a description of a method to cross-calibrate the well counter to the PET scanner as an add-on to procedures used to accredit sites for cross-calibration between PET scanner and dose calibrator, which can be obtained by following the EANM/EARL guidelines [15]. A previous study comparing the IDBC from the left ventricle with sampled blood showed a lower mean bias for ^89^Zr-studies of 0.2% [12]. The difference in bias might be because the cross-calibration procedure used in their study ensured that activity concentrations obtained with PET were accurate within 5% [12]. The origin of (remaining) bias (after cross-calibration of the well counter and dose calibrator) may be explained by several reasons. Firstly, increased noise levels in combination with low activity concentrations may result in a positive bias, due to the non-negativity constraint [16]. Secondly, scatter from neighbouring structures with high radioactivity may influence the bias, for example, scatter from the liver to the descending aorta and left ventricle would increase the bias. This provides the potential for residual, region and scanner dependent bias, even after cross-calibration of the various measurement systems.

The biological half-life could be estimated from any blood pool region within 11% bias, where the ascending and descending aorta showed the lowest bias of only 1–7%. The half-lives of ^89^Zr-cetuximab are comparable to previously reported biological half-lives of cetuximab of 70–208 h for a 500 mg/m^2^ dose [17]. For durvalumab, a half-life of 408 h was previously reported for a therapeutic dose of 10 mg/kg [18]. The lower half-lives for ^89^Zr-durvalumab in the current study are likely due to of the lower administered mass dose. It is important to note that the half-lives in this study were calculated using a limited amount of data, only for the purpose of comparing sample-derived and image-derived estimations. However, the results suggest that deriving an image-derived whole blood half-life can be useful in the assessment of human dosimetry in early stage clinical trials. Using the guidelines of EANM, red marrow residence times are calculated based on whole blood activity concentrations [19, 20]. Dosimetry calculations based on SDBC and IDBC give comparable results, with a bias on the red marrow effective dose of 2.4% and on the total body effective dose (mSv/MBq) of 0.3% when using the IDBC from the ascending aorta (see Additional file 1: SM3).

This study shows that blood samples may be replaced by IDBC from the ascending aorta. This is in line with previous studies, in which the ascending aorta was also the preferred region to obtain IDBC [7, 8, 21]. For the more advanced long axial field of view PET scanners, this study should be repeated as improved accuracy and precision can be expected [22].

## Conclusion

Image-derived blood concentrations should be obtained from delineating the ascending aorta in ^89^Zr-immuno-PET scans, as this results in the lowest variability with respect to sample-derived blood concentrations. To better understand the observed bias a cross-calibration between PET scanner and well counter should be performed.

### Supplementary Information

**Additional file 1: **Additional methods, results and analyses on how to obtain the image-derived blood concentration from ^89^Zr-immuno-PET scans.

## Data Availability

All data analysed during this study are available from the corresponding author on reasonable request.

## Abbreviations

EGFR: Epidermal growth factor receptor
IDBC: Image-derived blood concentration
IDIF: Image-derived input function
PD-L1: Programmed death ligand 1
PET: Positron emission tomography
PK: Pharmacokinetics
SD: Standard deviation
SUV: Standardized uptake value
TBR: Tumour-to-blood ratio
^89^Zr: Zirconium-89
