# Supplementary material for: How to obtain the image-derived blood concentration from 89Zr-immuno-PET scans
Source: EJNMMI Phys. 2024 Feb 7;11:16. doi: 10.1186/s40658-024-00621-7 (PMC10847076; doi:10.1186/s40658-024-00621-7)
Supplement: Supplementary file 1 — Additional file 1: Additional methods, results and analyses on how to obtain the image-derived blood concentration from 89Zr-immuno-PET scans. [file 40658_2024_621_MOESM1_ESM.docx]

How to obtain the image-derived blood concentration from ^89^Zr-immuno-PET scans

Jessica E. Wijngaarden^1,2^, Amina Ahbari^1,2^, Johanna E. E. Pouw^2,3^, Henri N. J. M. Greuter^1,2^, Idris Bahce^2,4^, Gerben J. C. Zwezerijnen^1,2^, Daniëlle J. Vugts^1,2^, Guus A. M. S. van Dongen^1,2^, Ronald Boellaard^1,2^, C. Willemien Menke-van der Houven van Oordt^2,3^, Marc C. Huisman^1,2^

**Author’s affiliations**^1^ Amsterdam UMC location Vrije Universiteit Amsterdam, Department of Radiology and Nuclear Medicine, Boelelaan 1117, Amsterdam, The Netherlands
^2^ Cancer Center Amsterdam, Imaging and Biomarkers, Amsterdam, The Netherlands
^3^ Amsterdam UMC location Vrije Universiteit Amsterdam, Department of Medical Oncology, Boelelaan 1117, Amsterdam, The Netherlands
^4^ Amsterdam UMC location Vrije Universiteit Amsterdam, Department of Pulmonary Medicine, Boelelaan 1117, Amsterdam, The Netherlands

**First and corresponding author:**Name: Jessica E. Wijngaarden
E-mail: j.e.wijngaarden@amsterdamumc.nlj.e.wijngaarden@amsterdamumc.nl
Address: De Boelelaan 1117, 1081 HV Amsterdam, The Netherlands
Telephone number: 020-4442863
Fax number: 020-4444329
ORCID: https://orcid.org/0000-0003-1486-6246

Supplemental figures:


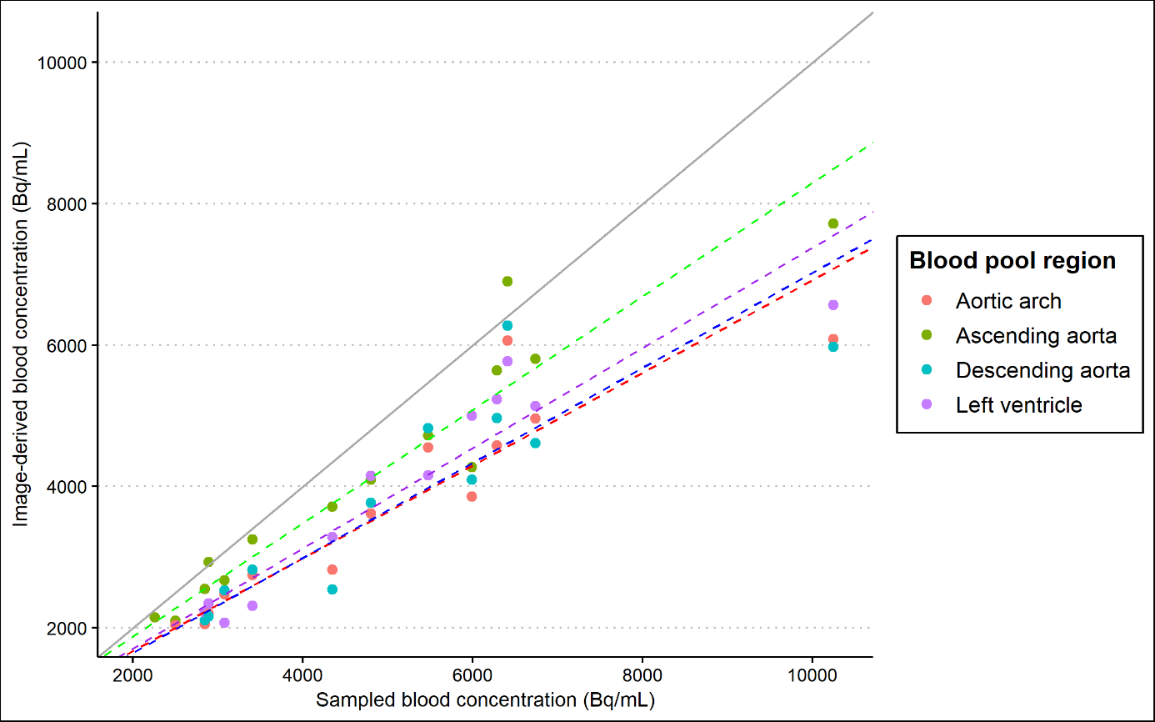
 **Figure S1**: Regression plots of image derived blood concentration versus sampled blood concentration for ^89^Zr-cetuximab. Four blood pool regions are indicated by different colors. The grey line indicates the line of identity.


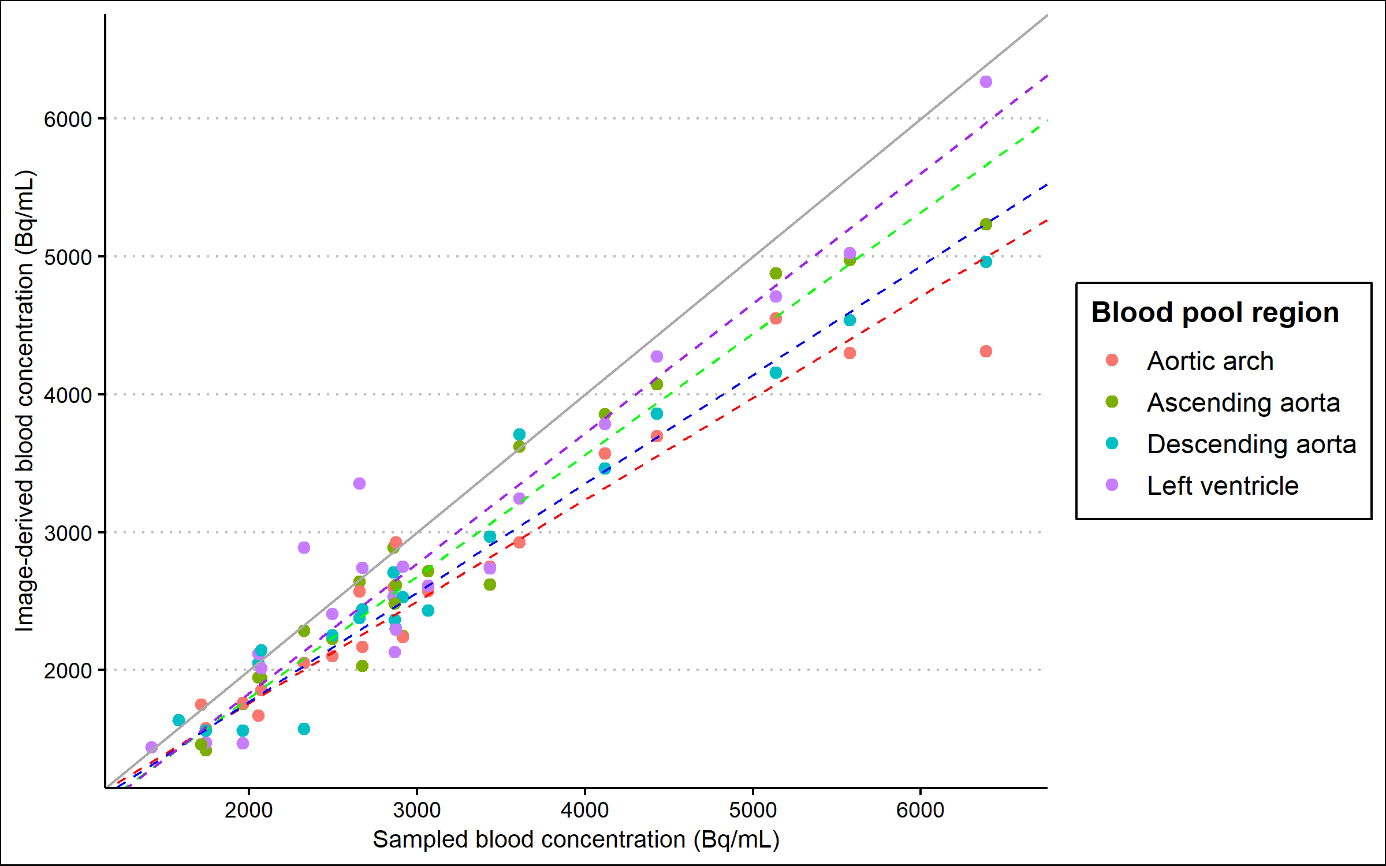

**Figure S2**: Regression plots of image derived blood concentration versus sampled blood concentration for ^89^Zr-durvalumab. Four blood pool regions are indicated by different colors. The grey line indicates the line of identity.

**Supplemental Table S1**: Comparison between half-life obtained from IDBC and from SDBC.

| Blood pool region | Mean half-life [h] from SDBC | Mean half-life [h] from IDBC | Mean percentage bias (%) | 1.96 times standard deviation (%) |
| --- | --- | --- | --- | --- |
| **^89^Zr-cetuximab** | 69.7 |  | | |
| Aortic arch |  | 77.1 | 9.36 | 22.7 |
| Ascending aorta |  | 74.5 | 6.63 | 20.4 |
| Descending aorta |  | 72.0 | 2.91 | 12.7 |
| Left ventricle |  | 74.8 | 7.74 | 27.2 |
| **^89^Zr-durvalumab** | 123.3 |  | | |
| Aortic arch |  | 139.3 | 10.9 | 26.7 |
| Ascending aorta |  | 125.4 | 1.28 | 35.8 |
| Descending aorta |  | 127.7 | 2.86 | 34.8 |
| Left ventricle |  | 116.0 | -3.16 | 31.5 |

Supplemental material SM1:

**Delineation protocol image derived blood concentration**

**General settings:**

- Delineation was done using a tool developed by RB. The tool is a variant of the analysis software tool ACCURATE (developed in IDL version 8.4 (Harris Geospatial Solutions, Bloomfield, USA)), because it is geared towards delineation of organs instead of tumours.
- PET images were inversed for better visualization, where higher activity concentrations are lighter and lower activity concentrations are darker.

**Ascending and descending aorta (figure 1)**

1. Select the axial view
2. Delineation was done based on the low dose CT (ldCT)
3. Place a region of interest (ROI) of size 0.576 mL in the center of the lumen
4. Repeat step 3 for at least five and up to ten consecutive axial slices, until the end of the structure.
5. For most cranial delineation, choose the axial slice where the ascending and descending aorta are circular shaped (positioned perpendicular to the axial view).
6. For the most caudal delineated axial slice, ensure that the ROI is not placed within the heart by checking on the coronal view.
7. Check whether the delineation overlaps with the activity concentration on the PET. If not, replace the ROI within the lumen on the ldCT overlapping with the activity on the PET.


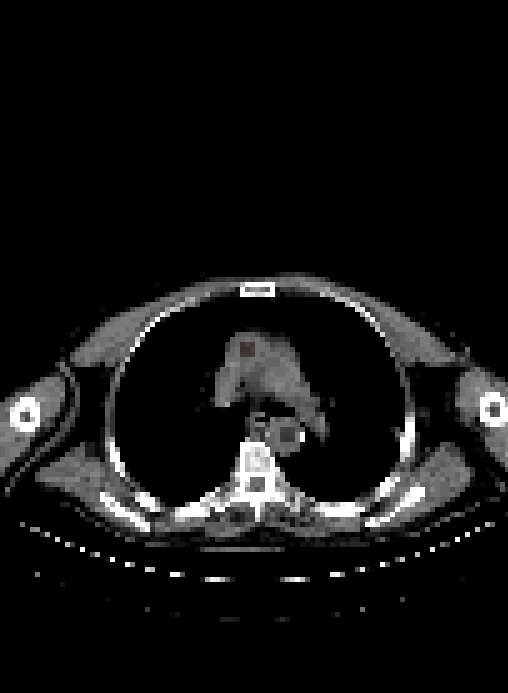

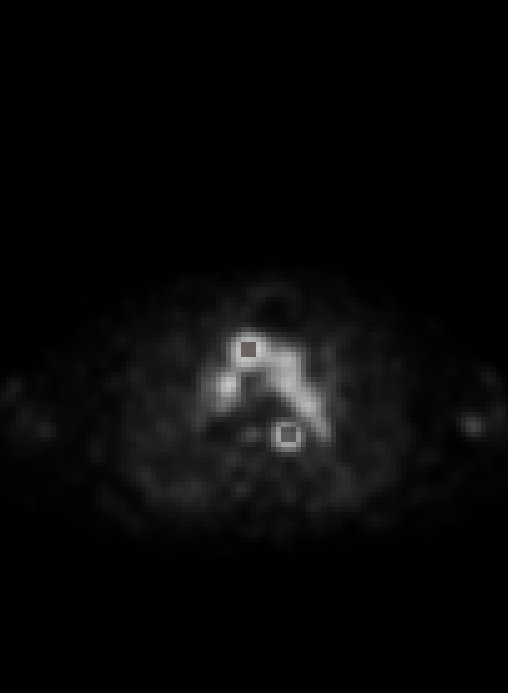


Figure 1: Axial view of the ascending and descending aorta delineation on the most cranial slice, with the ldCT on the left and the PET on the right.

**Aortic arch (figure 2)**

1. Select the axial view
2. Delineation was done based on the low dose CT (ldCT)
3. Delineate the aortic arch on two consecutive axial slices.
4. For the first delineation of the aortic arch, select the most caudal axial slice on which the ascending and descending aorta are fully connected. Place four ROIs of size 0.576 mL along the course of the aortic arch within that axial slice.
5. Repeat step 4 on the adjacent axial slice cranially located from the first delineated slice.
6. The whole delineation has a total volume of 4.6 mL.
7. Check whether the delineation overlaps with the activity concentration on the PET. If not, replace the ROI within the lumen on the ldCT overlapping with the activity on the PET.


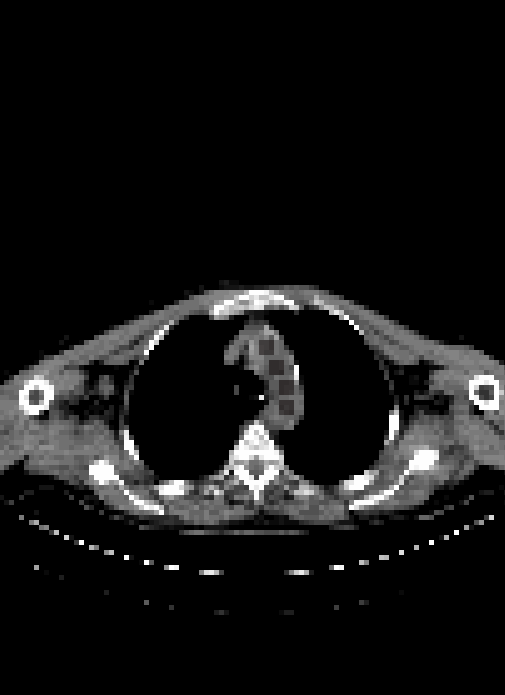

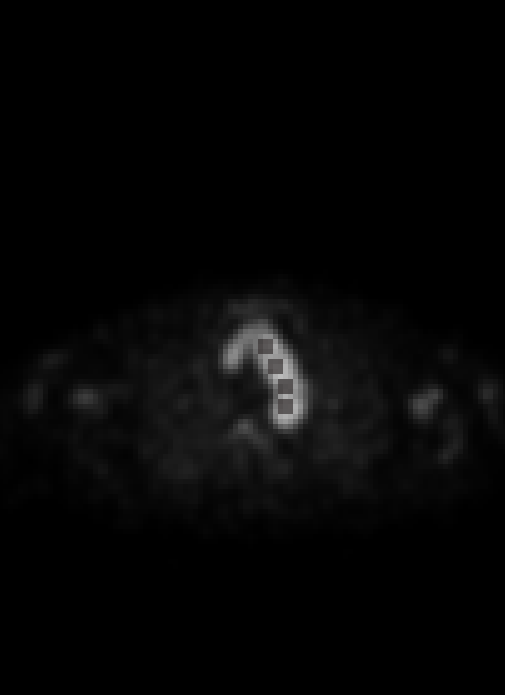


Figure 2: Axial view of the aortic arch delineation on the most cranial slice, with the ldCT on the left and the PET on the right.

**Left ventricle (figure 3)**

1. Select the axial view
2. Delineation was done based on the low dose CT (ldCT)
3. The ROI was placed in the center of the left ventricle.The location was chosen based on adjacent structures.
4. Repeat step 3 three times on three consecutive axial slices. The whole delineation has a total volume of 1.7 mL
5. Check whether the delineation overlaps with the activity concentration on the PET. If not, replace the ROI within the lumen on the ldCT overlapping with the activity on the PET.


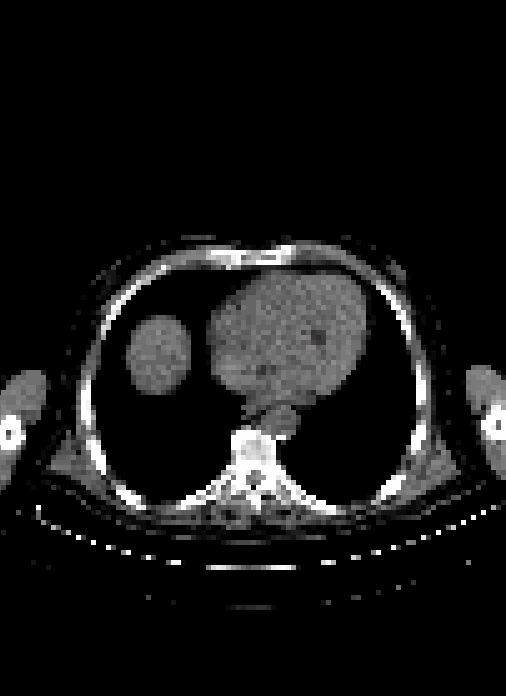

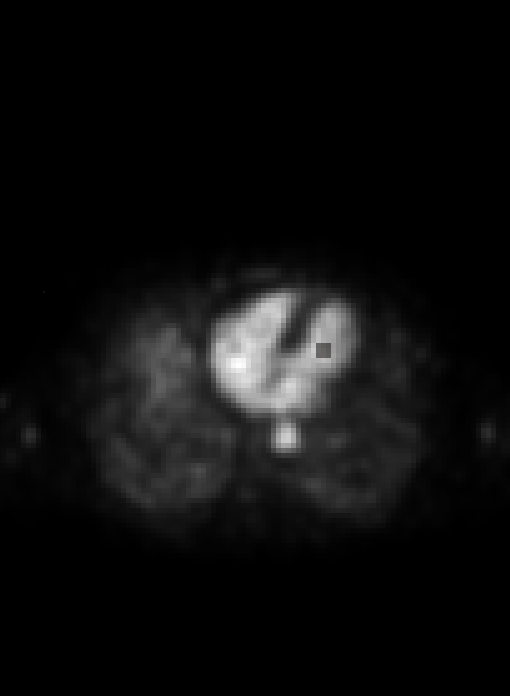


Figure 3: Axial view of the left ventricle delineation, with the ldCT on the left and the PET on the right.

Supplemental material SM2:

**Well counter cross-calibration protocol**Add on to the EARL cross-calibration standard operating procedure

**Definitions:**

- IT = injection time and date (hh:mm:ss on yyyymmdd)
- RT = reference time and date of PET acquisition, determined at production (hh:mm:ss on yyyymmdd)
- WCT = well counter acquisition time and date (hh:mm:ss on yyyymmdd)

**Materials:**

1. Calibration solution of 60 to 70 MBq Zirconium-89 (aimed at RT)
2. Cylindrical calibration phantom
3. PET/CT system
4. Dose calibrator
5. Well counter to be cross-calibrated

**Methods:**

1. Prior to the experiment, create an acquisition protocol for each well counter that consists of a 5 minute acquisition with an open energy window (e.g. 400-1100 keV). Measurements in cpm/g should be obtained.
2. Follow the EANM/EARL guidelines for ^89^Zr PET/CT Calibration QC (Kaalep et al., 2018). In summary, a calibration solution of 60 to 70 MBq ^89^Zr is added to the water-filled phantom. Thereafter, the phantom is scanned with the PET/CT system.
3. After completion of the EARL accreditation procedure, take three 0.5 mL samples from the phantom.
4. Determine the weight of each sample using a calibrated balance (should be about 0.5 grams).
5. Measure the samples in a well counter, note the cpm, time and date at WCT.
6. Combine the weight of the samples and the number of counts per minute, decay corrected for the time difference between IT and WCT, and note the resulting well counter rate concentration, R_wc_ (cpm/g) of the samples at IT.

**Example calculations:**

Per sample, the well counter measured counts in cpm are divided by the weight of the sample to obtain the concentration of counts in cpm/g at WCT. Decay correction is applied to obtain the well counter rate concentration (R_wc_) in cpm/g at IT.

On the PET scan, a volume of interest is delineated in the center of the phantom from which the average activity concentration can be derived.

The activity concentration in the phantom based on the PET images (AC_PET_) is divided by the average of the three R_wc_ values to obtain the cross-calibration factor (CC):

$$CC= \frac{{AC}_{PET}}{\frac{\sum R_{wc}}{3}}$$

This cross-calibration factor can be used to adjust the well counter measurements to the PET system.

| Measurement | | Value | Units | Value | Units | Time and date | | |
| --- | --- | --- | --- | --- | --- | --- | --- | --- |
| 1 | Activity syringe measured with dose calibrator (A_DC_): | 52.39 | MBq |  |  | @ time:  date: | 13:37:00 20230331 | IT |
| 2 | Residual activity in syringe (A_res_): | 0.238 | MBq |  |  | @ time:  date: | 13:37:00 20230331 | IT |
|  |  |  |  |  |  |  |  |  |
| 3 | Sample 1: | 0.498 | g | 88125 | cpm | @ time:  date: | 18:03:00 20230331 | WCT |
| 4 | R_wc_ | 184030 | cpm/g |  |  | @ time:  date: | 13:37:00 20230331 | IT |
| 5 | Sample 2: | 0.506 | g | 91441 | cpm | @ time:  date: | 18:09:00 20230331 | WCT |
| 6 | R_wc_ | 188102 | cpm/g |  |  | @ time:  date: | 13:37:00 20230331 | IT |
| 7 | Sample 3: | 0.523 | g | 95483 | cpm | @ time:  date: | 18:14:00 20230331 | WCT |
| 8 | R_wc_ | 190173 | cpm/g |  |  | @ time:  date: | 13:37:00 20230331 | IT |

Supplemental material SM3:

**Dosimetry analyses using different whole blood concentration measurements**

Average residence times for the ^89^Zr-cetuximab study are 1.28 h in kidney, 21.9 h in liver, 2.13 h in lungs, 0.71 h in spleen (Makris et al., 2015). The residence time of red marrow can be calculated as:

0.34*residence time of whole blood (Hindorf et al., 2010; Makris et al., 2015).

For this, we calculated the residence time of whole blood based on the two methods, sample derived (SD) and image derived (ID, based on the four regions).

The average whole blood residence time can be calculated as:

Residence time = 1.443 · T_1/2,eff_ (Loevinger et al., 1988)

With the effective half-life calculated as:

$\frac{1}{T_{1/2,eff}}= \frac{1}{T_{1/2,bio}}$ +$\frac{1}{T_{1/2,phys}}$

With the biological half-lives based on SDBC (69.7 h) and IDBC as presented in the supplemental table S1. And physical half-life is 78.41 h for ^89^Zr.

We subtract the residence times of kidney, liver, lungs, spleen and red marrow from the theoretical total residence time of 113 h (assuming no excretion from the body) to obtain the residence time of remainder of body (ROB).

We used the following as input to OLINDA/EXM (Stabin 1996): residence times for organs (1.28 h in kidney, 21.9 h in liver, 2.13 h in lungs, 0.71 h in spleen, ROB), total residence time of 113 h and the red marrow residence time for the different methods (presented in the table above in row nr. 3).
The results of red marrow effective dose and total body dose are obtained from OLINDA/EXM.

|  | **Sample** | **Aortic arch** | **Ascending aorta** | **Descending aorta** | **Left ventricle** |
| --- | --- | --- | --- | --- | --- |
| Whole blood biological half-life (h) | 69.7 | 77.1 | 74.5 | 72.0 | 74.8 |
| Whole blood residence time (h) | 53.2 | 56.1 | 55.1 | 54.2 | 55.2 |
| Red marrow residence time (h) | 18.1 | 19.1 | 18.7 | 18.4 | 18.8 |
| Red marrow effective dose (mSv/MBq) | 0.123 | 0.128 | 0.126 | 0.125 | 0.126 |
| Total body effective dose (mSv/MBq) | 0.604 | 0.608 | 0.606 | 0.605 | 0.607 |
| Relative difference in red marrow effective dose compared to sample-derived | 0% | 4.07% | 2.44% | 1.63% | 2.44% |
| Relative differences in total body effective dose compared to sample-derived | 0% | 0.66% | 0.33% | 0.17% | 0.50% |

We compared the total body dose between the different whole blood methods. The percentage differences (presented in the table above) are calculated per image-derived method as:

%diff = (ID-SD)/SD*100%

Table S2: Dosimetry analyses using different methods to quantify whole blood activity concentration.

Dosimetry calculations based on SDBC and IDBC give comparable results, with a bias on the red marrow effective dose of 2.4% and on the total body effective dose (mSv/MBq) of 0.3% when using the IDBC from the ascending aorta.

**References**

Hindorf, C., Glatting, G., Chiesa, C., Lindén, O., & Flux, G. (2010). EANM Dosimetry Committee guidelines for bone marrow and whole-body dosimetry. *European journal of nuclear medicine and molecular imaging*, *37*, 1238-1250.

Loevinger, R., Budinger, T. F., & Watson, E. E. (1988). MIRD primer for absorbed dose calculations. *(No Title)*.

Makris, N. E., Boellaard, R., van Lingen, A., Lammertsma, A. A., van Dongen, G. A., Verheul, H. M., ... & Huisman, M. C. (2015). PET/CT-derived whole-body and bone marrow dosimetry of 89Zr-cetuximab. *Journal of Nuclear Medicine*, *56*(2), 249-254.

Stabin MG. MIRDOSE: personal computer software for internal dose assessment in nuclear medicine. J Nucl Med. 1996 Mar;37(3):538-46. PMID: 8772664.
